# The *PEG13*-DMR and brain-specific enhancers dictate imprinted expression within the 8q24 intellectual disability risk locus

**DOI:** 10.1186/1756-8935-7-5

**Published:** 2014-03-25

**Authors:** Franck Court, Cristina Camprubi, Cristina Vicente Garcia, Amy Guillaumet-Adkins, Angela Sparago, Davide Seruggia, Juan Sandoval, Manel Esteller, Alex Martin-Trujillo, Andrea Riccio, Lluis Montoliu, David Monk

**Affiliations:** 1 Imprinting and Cancer Group, Cancer Epigenetics and Biology Program (PEBC), Bellvitge Institute for Biomedical Research (IDIBELL), L’Hospitalet de Llobregat, Barcelona 08907, Spain; 2 Department of Molecular and Cellular Biology, Centro Nacional de Biotecnología (CNB-CSIC), Madrid 28049, Spain; 3 CIBERER-ISCIII, Madrid 28049, Spain; 4 Institute of Genetics and Biophysics, A Buzzati-Traverso, CNR, Naples 81031, Italy; 5 Cancer Epigenetics Group, Cancer Epigenetics and Biology Program (PEBC), Bellvitge Institute for Biomedical Research (IDIBELL), L’Hospitalet de Llobregat, Barcelona 08907, Spain; 6 Department of Physiological Sciences II, School of Medicine, University of Barcelona, Barcelona, Catalonia, Spain; 7 Institucio Catalana de Recerca i Estudis Avançats (ICREA), Barcelona, Catalonia, Spain; 8 Current address: Unidad de Genética Médica, Sistemas Genómicos SL, Paterna, Valencia 46980, Spain

**Keywords:** Imprinting, DNA methylation, Chromatin looping

## Abstract

**Background:**

Genomic imprinting is the epigenetic marking of genes that results in parent-of-origin monoallelic expression. Most imprinted domains are associated with differentially DNA methylated regions (DMRs) that originate in the gametes, and are maintained in somatic tissues after fertilization. This allelic methylation profile is associated with a plethora of histone tail modifications that orchestrates higher order chromatin interactions. The mouse chromosome 15 imprinted cluster contains multiple brain-specific maternally expressed transcripts including *Ago2*, *Chrac1*, *Trappc9* and *Kcnk9* and a paternally expressed gene, *Peg13*. The promoter of *Peg13* is methylated on the maternal allele and is the sole DMR within the locus. To determine the extent of imprinting within the human orthologous region on chromosome 8q24, a region associated with autosomal recessive intellectual disability, Birk-Barel mental retardation and dysmorphism syndrome, we have undertaken a systematic analysis of allelic expression and DNA methylation of genes mapping within an approximately 2 Mb region around *TRAPPC9.*

**Results:**

Utilizing allele-specific RT-PCR, bisulphite sequencing, chromatin immunoprecipitation and chromosome conformation capture (3C) we show the reciprocal expression of the novel, paternally expressed, *PEG13* non-coding RNA and maternally expressed *KCNK9* genes in brain, and the biallelic expression of flanking transcripts in a range of tissues. We identify a tandem-repeat region overlapping the *PEG13* transcript that is methylated on the maternal allele, which binds CTCF-cohesin in chromatin immunoprecipitation experiments and possesses enhancer-blocker activity. Using 3C, we identify mutually exclusive approximately 58 and 500 kb chromatin loops in adult frontal cortex between a novel brain-specific enhancer, marked by H3K4me1 and H3K27ac, with the *KCNK9* and *PEG13* promoters which we propose regulates brain-specific expression.

**Conclusions:**

We have characterised the molecular mechanism responsible for reciprocal allelic expression of the *PEG13* and *KCNK9* transcripts. Therefore, our observations may have important implications for identifying the cause of intellectual disabilities associated with the 8q24 locus.

## Background

Genomic imprinting is the epigenetic marking of a subset of genes that results in parent-of-origin monoallelic expression. The regulation of imprinting is complex, involving interplay between many different epigenetic mechanisms, including DNA methylation, histone tail modifications and non-coding RNA (ncRNAs) [1]. Most imprinted genes are associated with a region of differential DNA methylation that is acquired in the gametes, and maintained in somatic tissues after fertilization by the UHRF1-DNMT1 complex [2-4].

The majority of imprinted differentially DNA methylated regions (DMRs) acquire their methylation from oocytes, as only a few examples of sperm-derived methylation at DMRs are known [5]. Maternally methylated DMRs generally act as promoters, associated in some cases with long ncRNAs. In some cases these ncRNAs confer silencing of neighbouring genes in *cis* through recruitment of histone remodelling complexes [6,7]. Some of the intergenic, paternally methylated, DMRs act as methylation-sensitive insulators recruiting CTCF [8], influencing higher-order chromatin folding [8].

**Figure 1:**
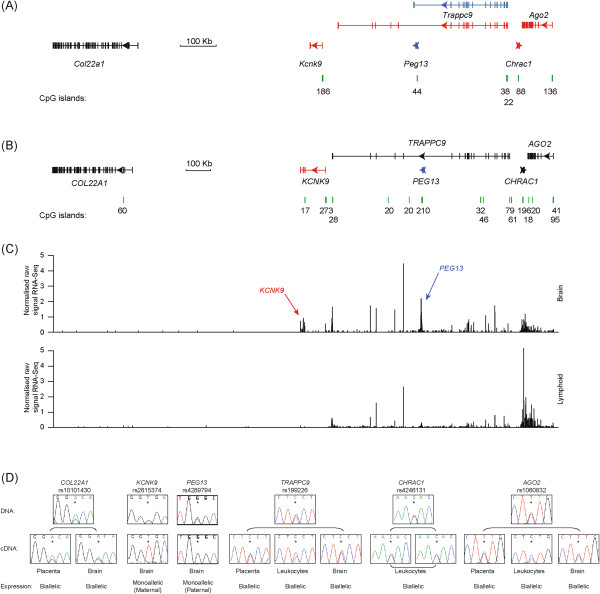
**Schematic representation of the *Peg13*/*PEG13 *imprinted domain on distal mouse chromosome 15 and human chromosome 8q24. (A)** Map of the *Trappc9/Peg13* locus located on mouse chromosome 15, showing the location of the various imprinted transcripts (red transcripts are maternally expressed, blue are paternally expressed and black are expressed from both parental alleles; arrows represent direction of transcription) and CpG islands (green bars). **(B)** Schematic of the human *TRAPPC9/PEG13* region on chromosome 8. **(C)** RNA-seq profiles throughout the human domain in brain and lymphoid tissue. The RNA peaks corresponding to *PEG13* and *KCNK9* are indicated in the brain track. The RNA density plots represent relative read density (normalised counts per million mapped reads as defined by the University of California Santa Cruz (USCS) sequence browser). **(D)** The allelic expression for the genes in the human cluster. The sequence traces for heterozygous DNA samples from brain, leucocytes and term placentae are shown, as well as the resulting RT-PCR.

Aberrant imprinting in the brain is known to be associated with severe developmental disorders such as Angelman and Prader-Willi syndromes, and other behavioural phenotypes including autism-spectrum disorder (ASD), mental retardation (MR) and psychosis [9,10]. Recently, several genome-wide scans have identified susceptibility alleles for MR and ASD mapping to 8q22-24 [10]. This approach was used to identify maternally inherited missense mutations of the imprinted two pore-domain potassium channel (K2P) gene, *KCNK9* (also known as *TASK3*) in Birk-Barel mental retardation syndrome [11]. This gene is located adjacent to *TRAPPC9* (also known as *NIBP*), a gene disrupted by both coding mutations and deletions in non-syndromic, autosomal-recessive mental retardation [12-15]. The orthologous region on mouse chromosome 15 shows parent-of-origin allelic expression in mouse brain, and includes the maternally expressed *Kcnk9*[16], *Trappc9*, *Chrac1* and *Ago2* (also known as *Eif2c2*) genes, clustered around the paternal *Peg13* ncRNA (Figure 1A).

In the present study, we characterise the human 8q24 locus, and show that only *PEG13* and *KCNK9* are imprinted (Figure 1B). These transcripts are reciprocally expressed, with paternal expression of *PEG13* and maternal expression of *KCNK9*. The *PEG13* transcript is embedded within a maternal methylated region, with the unmethylated allele enriched for CTCF-cohesin, which acts as an enhancer-blocker *in vivo*. Lastly, we show chromatin loops between an enhancer region, with a canonical enhancer histone modification signature in brain tissues, and the *KCNK9* and *PEG13* promoters.

## Results

### Imprinting within human chromosome 8q24

A 700 kb region on mouse chromosome 15 was recently identified as a novel imprinted domain using ultra-sensitive RNA-seq technology [17]. The cluster of four maternally expressed genes, as well as numerous expressed sequence tags (ESTs) is located around the paternally expressed 4.7 kb ncRNA, *Peg13*, within intron 17 of the *Trappc9* gene. The *Trappc9* gene itself is subject to isoform-specific imprinting, with a single truncated isoform being paternally expressed, however, all the full-length transcripts are maternally derived (Figure 1A) [17]. The promoter of *Peg13* is a CpG island (CpG44 in Figure 1A) that is methylated on the maternal allele, being established in developing oocytes [18,19].

To assess if imprinting is conserved within the human orthologous region, we assessed the allelic expression for *COL22A1, KCNK9, TRAPPC9, CHRAC1* and *AGO2* (Figure 1B). We identified transcribed single nucleotide polymorphisms (SNPs) that would allow for allelic discrimination, and imprinting analysis was performed in adult leucocytes, brain and term placentae. We confirm that *KCNK9* is monoallelically expressed in the majority of brain regions, from the maternal allele (n = 2; including whole brain, hippocampus, cerebellum, vermis, parietal lobe and entorhinal cortex). RT-PCR followed by pyrosequencing confirmed the Sanger sequencing results and revealed that only residual expression arises from the repressed allele (Additional file 1: Figure S1). All other transcripts were expressed biallelically in multiple tissues (Figure 1D; Additional file 2: Table S2).

### The human *PEG13* ncRNA is comprised of numerous expressed tandem-repeats

We identified numerous ESTs (reference AX748239), the majority derived from the cerebellar and amygdalar regions of the brain, overlapping a large CpG island within intron 17 of the human *TRAPPC9* gene (CpG210 in Figure 1B), which is in the orthologous intron as the mouse *Peg13* gene. Sequence analysis revealed no similarity with mouse *Peg13*, despite their similar location. The region is made up of numerous degenerative simple tandem repeats, including 4.7 copies of a 96 bp repeat and 43.5 copies of a 15 bp repeat. Despite numerous attempts, we failed to detect a signal by northern blot analysis, however this region is associated approximately 6 kb of contiguous overlapping RNA-seq reads in brain derived RNA (Burge lab brain and ENCODE RNA-seq track UCSC genome browser GRCh37/hg19) suggesting that expression across this repetitive region produces a single transcript (Figure 1C). Using qRT-PCR we were able to determine that the expression was most abundant in brain (Additional file 1: Figure S1) and, in samples heterozygous for rs4289794, was monoallelically expressed (n = 10; including cerebellum, vermis, hippocampus, parietal lobe and occipital cortex). Pyrosequencing assay confirmed these observations (Additional file 1: Figure S1). One sample was accompanied by informative parental DNA samples and so the paternal origin of the expressed allele was ascertained (Figure 1D).

### The CpG island overlapping *PEG13* is a maternally methylated DMR

**Figure 2:**
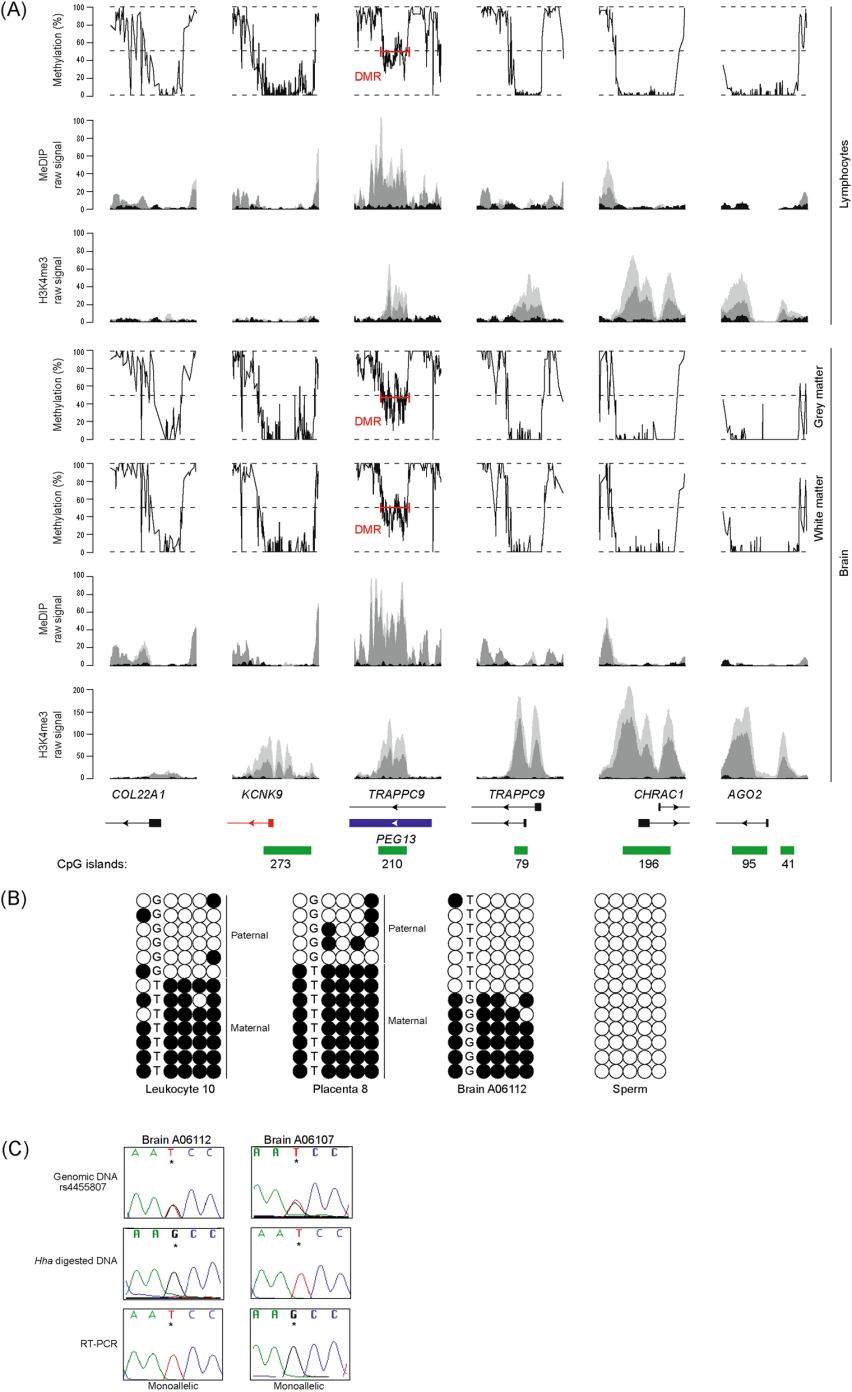
**DNA methylation profiling of gene promoter of genes flanking *TRAPPC9/PEG13*. (A)** CpG methylation profiling in lymphocytes and brain samples as defined by WGBS. The analyses were restricted to the intervals overlapping the promoters/CpG islands and are associated with the expected H3K4me3 and meDIP signatures in CD4 lymphocytes cells and fetal brain. The two shades of grey peaks in the meDIP and ChIP-seq panels represent two independent biological replicates compared to input (black peaks) with the y-axis showing the number of ChIP-seq reads. The precise location of the *PEG13*-DMR, as defined by approximately 50% methylation and co-enrichment of H3K4me3 and meDIP is indicated by the red bracket. **(B)** The methylation status of the *PEG13*-DMR was confirmed using standard bisulphite PCR on DNA samples heterozygous for SNP rs4455807. **(C)** Sequence traces for two brain samples revealing the genotypes of the *PEG13*-DMR methylated allele (resistant to *Hha*I digestion) and expression from the unmethylated allele.

It has previously been proposed that imprinting of this cluster in mouse is controlled by the maternally methylated germline DMR encompassing the promoter CpG island associated with the *Peg13*[18]. We analysed the DNA methylation status of all human promoter associated CpG islands throughout the domain in human leucocytes and brain tissues (grey and white matter isolated from the cerebral cortex, specifically the dorsolateral prefrontal cortex; Brodmann area 9) using a combination whole genome bisulphite sequencing (WGBS) and ChIP-seq for meDIP and H3K4me3, a histone modification refractory to DNA methylation. This high-throughput analysis revealed that the *KCNK9*, *AGO2*, *CHRAC1* and *TRAPPC9* promoter CpG islands are unmethylated in both tissue types and are associated with abundant H3K4me3 enrichment (Figure 2A). In addition, we observe that the CpG island overlapping *PEG13* is approximately 50% methylated in brain and lymphocyte WBGS datasets and is associated with co-enrichment of H3K4me3 and meDIP, consistent with differential active and repressive chromatin states on homologous chromosomes (Figure 2A). We subsequently analysed the methylation status of this region using bisulphite PCR and direct sequencing. This revealed that the *PEG13* region flanking the rs4455807 SNP exhibits allelic methylation in brain (whole fetal and adult cerebellum, hippocampus, frontal and occipital cortex), leucocytes and placenta-derived DNA (Figure 2B). In informative cases, the DMR was methylated on the maternal allele. In seven brain samples heterozygous for this polymorphism, we observed robust allelic methylation and monoallelic expression of the unmethylated allele, consistent with paternal expression (Figure 2C). One of these samples was directly shown to be paternally expressed when parental DNA samples were interrogated. Coherent with this observation, this interval is devoid of methylation in sperm derived DNA (Figure 2B).

### The paternal allele of the *PEG13*-DMR binds CTCF-cohesin

**Figure 3:**
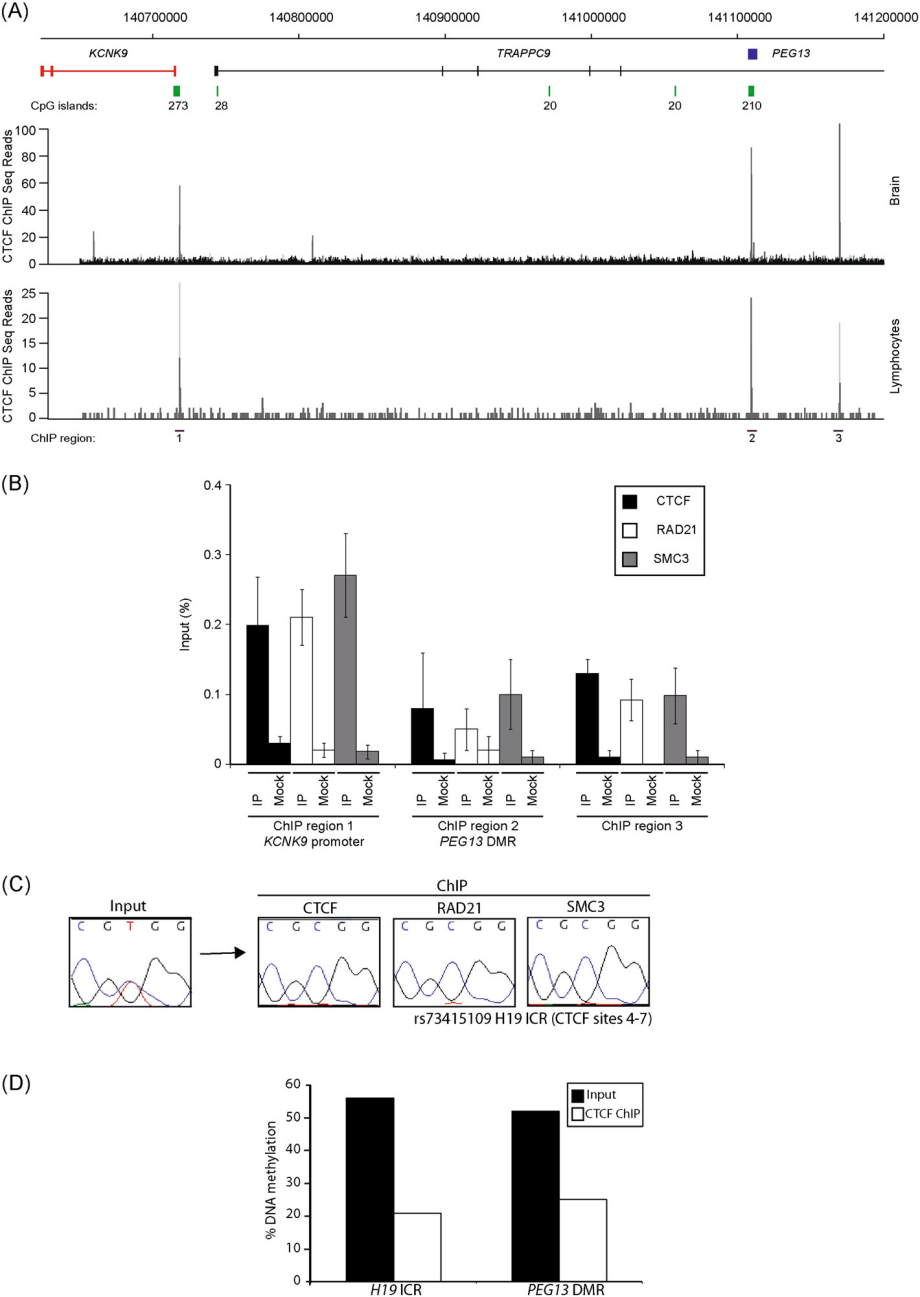
**ChIP analysis for CTCF and the cohesion subunits RAD21 and SMC3. (A)** ChIP-Seq data analysis in lymphocytes and cerebellum reveals the location of three ubiquitous CTCF binding sites. The positions of the ChIP PCRs are indicated. **(B)** qPCR performed on CTCF, RAD21 and SMC3 ChIP material in normal lymphoblastoid cells at the intervals identified by ChIP-Seq. Graphs are represented as % of precipitation relative to input chromatin (mean values ± SEM). **(C)** Sequence traces showing monoallelic precipitation of CTCF and cohesion subunits at the control *H19*-ICR. **(D)** The methylation levels of at the *H19*-ICR and *PEG13*-DMR in CTCF input and ChIP material as determined by bisulphite PCR followed by pyrosequencing.

Using a panel of DNA samples derived from various brain regions (parietal lobe, occipital lobe, cerebellum, frontal cortex, temporal gyrus, hippocampus and vermis) we confirm the unmethylated status of the promoter of *KCNK9*, which agrees with the observations obtained from WGBS (data not shown). This suggests that the imprinting of this gene is dependent on other *cis*-acting regulatory elements. The maternal expression of *KCNK9* and the maternal methylation of the *PEG13*-DMR are consistent with the action of an enhancer-blocker mechanism utilizing CTCF, similar to that which regulates the reciprocal imprinting of *Igf2/H19*[20]. Several strong canonical two-part CTCF (motif 1 + 2) binding sites within the *PEG13*-DMR were revealed using an *in silico* analysis using the published ChIP-sequence data (data not shown) [21,22]. To confirm *in vitro* binding, we investigated CTCF binding in published ChIP-seq datasets. We observed CTCF enrichment at the *PEG13*-DMR in brain and lymphocyte experiments and confirmed these observations utilizing ChIP on normal lymphoblast cells (region 2 of Figure 3). The efficiency of the ChIP was confirmed by parallel analysis of the *H19*-ICR, with maternal enrichment observed for a PCR amplicon incorporating CTCF sites 4 to 7 (Figure 3C) [23]. Subsequent analysis revealed precipitation of the *PEG13*-DMR. Unfortunately, the cell lines were not informative for any polymorphisms that would allow discrimination of parental alleles. To circumvent this, we performed bisulphite pyrosequencing for the *PEG13*-DMR on CTCF immunoprecipitated DNA. This revealed that, similar to the *H19*-ICR, CTCF preferentially binds to the unmethylated allele (Figure 2D). Recently, cohesin has been shown to play a critical role in maintaining CTCF higher-order chromatin confirmation at the *H19-IGF2* loci [8,24]. To determine if CTCF and cohesin co-localize at the *PEG13*-DMR, we performed ChIP using antisera against the cohesin subunits RAD21 and SMC3. In lymphoblast cell lines, the cohesin subunits clearly precipitated at the *PEG13*-DMR (Figure 3B), suggesting that a CTCF-cohesin complex may orchestrate higher order chromatin loops.

### The *PEG13*-DMR has enhancer-blocking

**Figure 4:**
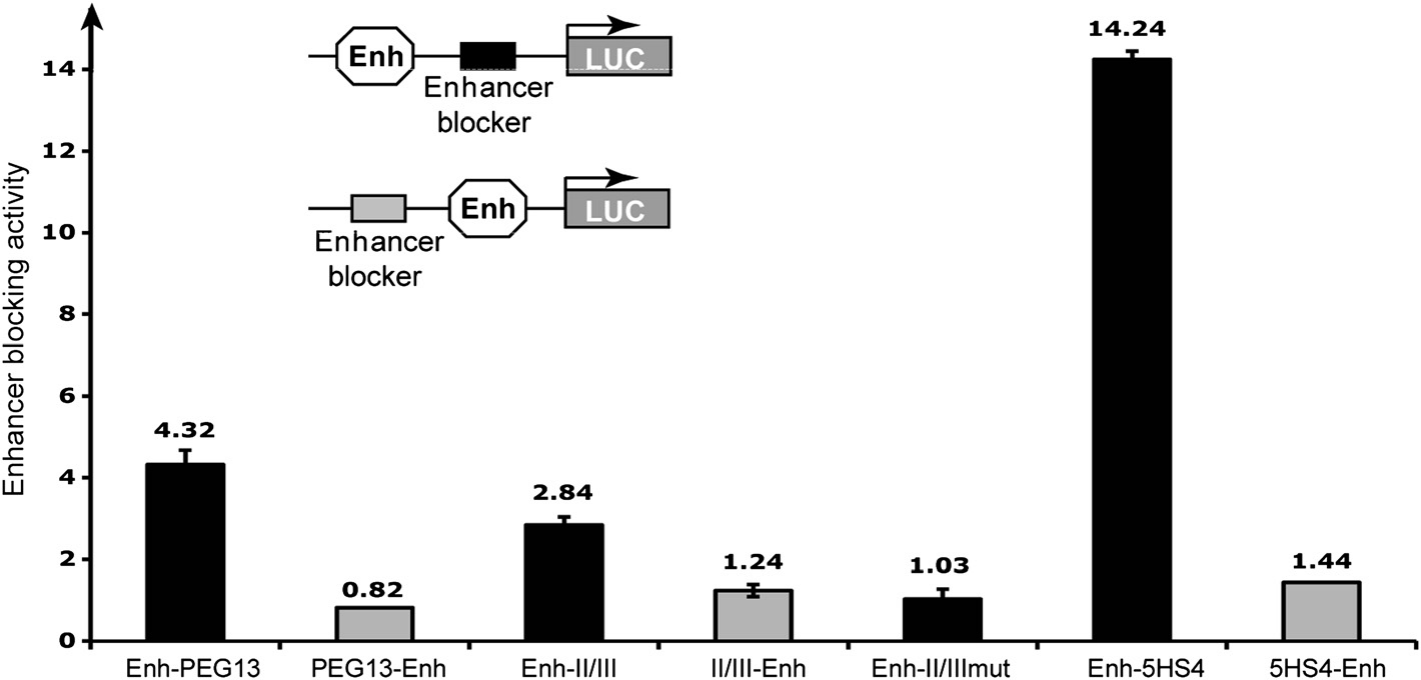
**The *PEG13*-DMR possesses insulator activity.** The bars indicate the firefly luciferase expression relative to *Renilla* luciferase activity for constructs containing a 480 bp fragment encompassing the *PEG13*-DMR. As a control, the enhancer-blocking assay was also performed with inserts for the 5HS4 (1.2 kb insulator) or the II/III (‘core’ 5′HS4) of β-globin enhancer-blocker. The II/III mut construct is the core 5′HS4 fragment with mutated CTCF sites that abolish insulator activity. The constructs are illustrated at the top of the figure. Data are presented as fold-enhancer-blocking activity normalised to the reference pELuc vector.

To test the *PEG13*-DMR for enhancer-blocking activity we used a previously described enhancer-blocking assay [25,26]. We studied the ability of a 480 bp *PEG13*-DMR fragment containing the canonical CTCF motifs to interfere with the activity of a heterologous enhancer/promoter interaction in HEK293 cells. The *PEG13*-DMR construct showed consistent enhancer-blocking activity, represented as fold reduction of activity compared to a vector lacking an insert (Figure 4). The *PEG13*-DMR fragment showed a stronger enhancer-blocking activity than the II/III control fragment containing the β-globin HS4 ‘core’ enhancer-blocker, but not as much as the 5HS4 insulator element.

### CTCF mediated allele-specific chromatin looping occurs between a shared enhancer and the *KCNK9* and *PEG13* promoters

**Figure 5:**
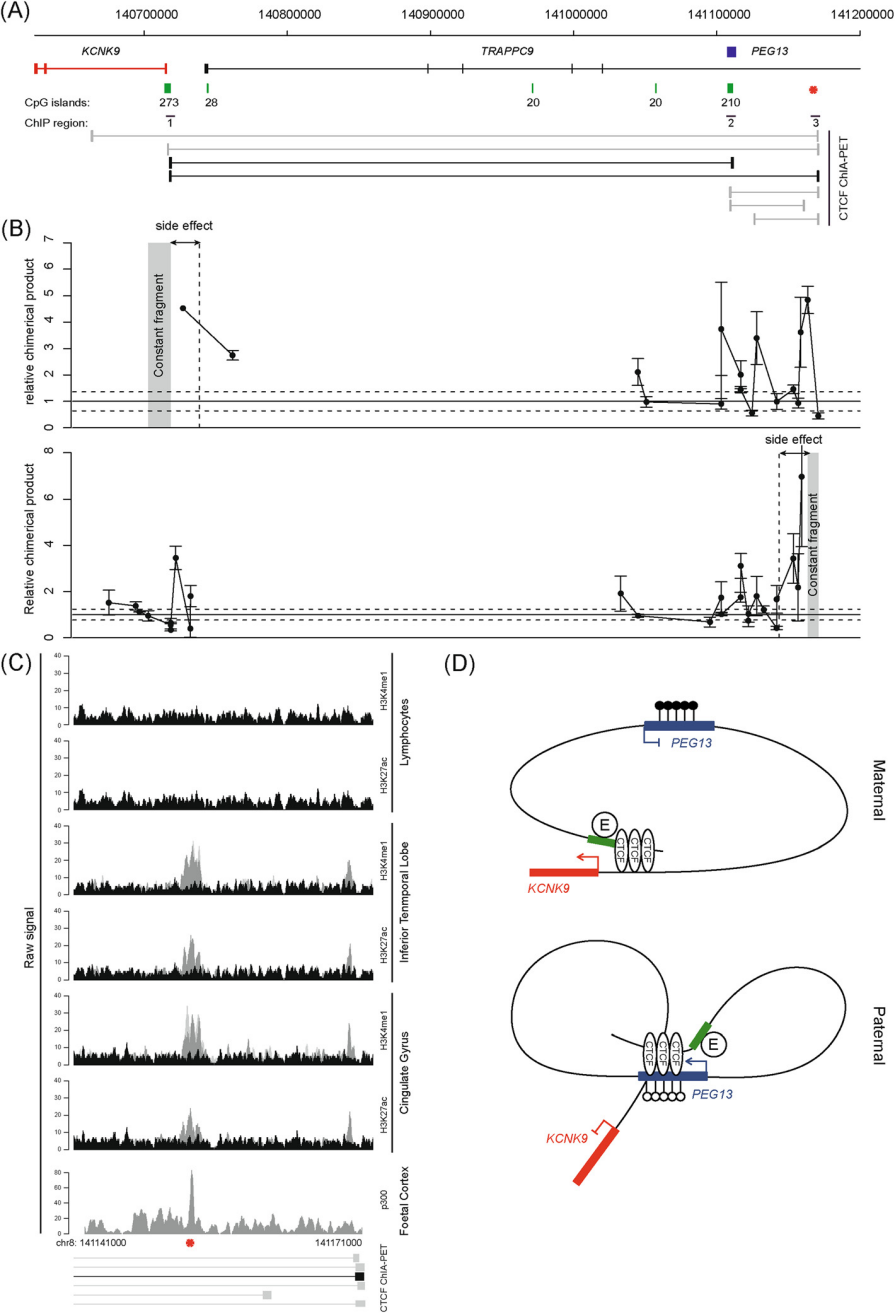
**Chromatin interactions with CTCF sites upstream of the *KCNK9 *promoter, the *PEG13*-DMR and a novel brain-specific enhancer. (A)** A schematic map of the *TRAPPC9* and *KCNK9* genes and annotated ChIA-PET data showing CTCF interactions. The red star represents the location of the brain-specific enhancer. **(B)** The 3C looping profile in brain using a constant primer 5′ to the *KCNK9* promoter (upper graph) or within the enhancer located within intron 17 of *TRAPPC9* (lower graph). **(C)** Sequence track for H3K4me1 and H3K27ac for a 30 kb region containing the *TRAPPC9* intron 17 ChIA-PET site. Tracks are shown in expressing in brain and non-neuronal lymphocyte samples. The two shades of grey peaks in the meDIP and ChIP-seq panels represent two independent biological replicates compared to input (black peaks) with the y-axis showing the number of ChIP-seq reads. The red star depicts the location of the proposed enhancer region. **(D)** A proposed model of higher-order chromatin looping within the 8q24 domain, showing the special organisation of the allele-specific *PEG13* enhancer-blocker in relation to the gene promoters, CTCF binding sites and H3K4me1/H3K27ac enhancer region. (●) methylated cytosine, (O) unmethylated cytosines and green block representing the H3K4me1/H3K27ac region associated with the brain-specific enhancer (e).

Recent studies have revealed that CTCF occupancy correlates with domain boundaries, as well as mediating intra- and interchromosomal contacts [27]. In an attempt to understand the mechanism regulating reciprocal brain-specific expression of *KCNK9* and *PEG13*, we interrogated publically available genome-wide ChIA-PET from MCF-7 cells and CTCF ChIP-seq datasets. We identified additional strong CTCF enrichment at the *KCNK9* promoter and a region within intron 17 of *TRAPPC9* (regions 1 and 3 of Figures 3 and 5C, respectively), which has an enhancer chromatin signature with the co-enrichment of H3K4me1, H3K27ac and p300 in various brain ChIP-seq datasets, including the frontal cortex [28,29]. Interestingly, in lymphocytes, which do not express *KCNK9* or *PEG13*, this region is not associated with either histone modification, suggesting that that it is a brain-specific enhancer (Figure 5C). Subsequent ChIP analysis confirmed that both of these regions were strongly enriched for both CTCF and the cohesin subunits RAD21 and SMC3 (Figure 3B), both of which had insulator function in our enhancer-blocking assay (Additional file 3: Figure S2). The ChIA-PET dataset revealed that these CTCF-cohesin regions, separated by approximately 500 kb, physically interact (Figure 5A).

To confirm this physical interaction, we performed chromatin conformation capture experiments (3C) to identify potential chromatin folding. 3C-qPCR assays were performed on cerebellar samples and interaction frequencies were determined between a constant *Hind*III site located within the unmethylated CTCF-cohesin binding site within the *KCNK9* promoter and other *Hind*III sites throughout the locus. We identified strong interactions between the *KCNK9* promoter constant fragment with the *PEG13*-DMR and the CTCF-cohesin site in the enhancer region located within intron 17 of *TRAPPC9* (Figure 5B). Direct sequencing of the chimerical products from brain revealed that the appropriate chimeric products result from the 3C ligations (data not shown). We subsequently used a PCR primer within the enhancer region as the constant, to not only confirm the physical interaction with the *KCNK9* promoter, but to also determine whether this region juxtaposes the *PEG13* promoter. Using this second constant fragment, we observed that the enhancer region physically interacts with both the *KCNK9* and *PEG13* in human cerebellar samples (Figure 5B). Unfortunately, no informative SNPs were identified within the vicinity of the *Hind*III sites associated with the enhancer that would allow us to determine the parental origin of the resulting chemical products. These results suggest that the brain-specific reciprocal imprinted expression of *PEG13* and *KCNK9* is dependent upon the active enhancer configuration and higher-order chromatin looping (Figure 5D).

## Discussion

We have characterised a new imprinted cluster on human chromosome 8q24, which harbours two brain-specific genes. We show that the promoter of the novel, paternally expressed, non-coding *PEG13* transcript arises from a maternally methylated DMR. This DMR binds a CTCF-cohesin complex that acts as a methylation-sensitive enhancer-blocker on the unmethylated paternal allele. Furthermore, we have utilized publically available ChIA-PET datasets to identify an enhancer region that was previously unknown to be associated with gene expression within the locus. The ChIA-PET technique combines both chromatin immunoprecipitation with 3C-type analyses for the direct analysis of chromatin interactions exclusively formed between sites bound by a given chromatin interacting protein, in our case CTCF, however it does not address whether the identified loops depend on the protein of interest. We confirm the ChIA-PET interactions using standard 3C assays in brain. Our data suggest that the brain-specific expression of the imprinted transcripts is directly due to the acquisition of an active enhancer chromatin signature. The genomic regions immediately flanking the 3C/ChIA-PET interacting region within intron 17 of *TRAPPC9* is marked in neuronal tissues by H3K4me1, a histone mark associated with many cell-type specific enhancers, abundant H3K27ac, with the acetyl group presumably deposited by the P300 acetyltransferase [30].

DNA methylation composition in brain is highly dynamic, being present in several forms. An oxidative derivative of 5-methylcytosine (5mC) called 5-hydroxymethylcytosine (5-hmC) accumulates in adult brain [31,32]. In addition, methylation in non-CpG context (mCH, where H is A, C or T) is also more abundant in frontal cortex of both adult mouse and humans [32]. Previous reports have shown that the adult brain contains high levels of 5-hmC, accounting for approximately 40% of CG methylation in cerebellar Purkinje cells [31]. This modification is especially enriched in multi-exonic, highly expressed brain genes [33]. Methodologies based on standard bisulphite-sequencing techniques cannot distinguish between abundant 5mC and 5-hmC and sites, so the methylation profiles we identified by WGBS or bisulphite PCR will represent the sum of both modifications. However, our results using meDIP using an antibody directed against 5mC strongly suggest that the methylation we observe is not 5-hmC. This observation at the brain-specific *PEG13* transcript is consistent with intronless, single exon genes having significantly less, or no, 5-hmC when compared to spliced transcripts [34]. In addition, a previous genome-wide study has identified ‘diffuse’ parent-of-origin dependent non-CG methylation sites overlapping the gene bodies of the mouse *Ago2* and *Peg13* genes [19]. Our analyses did not reveal any allelic methylation outside the CpG context at the *PEG13* or *AGO2* gene in WGBS datasets from human grey and white brain matter or by direct bisulphite PCR in adult frontal cortex (Additional file 4: Figure S3) [19]. This evolutionary discrepancy may partially explain the lack of imprinting of the *AGO2* gene in humans.

Changes in the accumulation of DNA methylation in neurons throughout early childhood has been implicated in learning and memory, as well as cognitive function [32,35]. Some Angelman syndrome cases, as well as autism and ASD, are associated with coding mutations and methylation defects at the imprinted *SNRPN* locus [9,10]*.* Interestingly, similar to the long-range looping we that we have identified for *KCNK9*, the PWS/AS-IC interacts with the *CHRNA7* gene, a distance of more than 5 Mb [36,37]. This higher-order chromatin interaction is associated with MeCP2, suggesting that this protein, like CTCF, is capable of orchestrating long-range chromatin organisation in the brain [37].

## Conclusions

We identified a maternally methylated region that binds CTCF-cohesin, possesses enhancer- blocker activity, which we hypothesize dictates mutually exclusive chromatin looping between a novel enhancer region and the promoters of the reciprocally imprinted *PEG13* and *KCNK9* transcripts. Our findings are of particular relevance, not only to those researchers working on the aetiology of intellectual disabilities, but also to those interested in tissue-specific transcriptional regulation, since we show that the essential chromatin scaffold of the domain is present in all tissues but that a brain-specific enhancer is the only regulatory element dictating tissue specificity. It will be interesting to determine whether the *PEG13* ncRNA functional contributes to the imprinting of the *KCNK9* gene and whether these two imprinted transcripts are subject to epigenetic deregulation of the *cis*-acting sequences that could silence or cause loss-of-imprinting in non-syndromic forms of intellectual disability.

## Methods

### Human tissues

A cohort of 48 placental samples with corresponding cord blood was collected at Hospital Sant Joan de Déu (Barcelona, Spain). DNA and RNA extraction and cDNA synthesis were carried out as previously described [38]. Normal peripheral blood was collected from adult volunteers aged between 19 and 60 years of age. A total of 40 normal adult brain samples were obtained from BrainNet Europe/Barcelona Brain Bank. The dissection of individual brain regions (hippocampus, cerebellum, vermis, parietal lobe, entorhinal and occipital cortices) was performed by an experienced pathologist on cadavers within 14 hours of death. The human RNA panel was purchased from Clontech (Human Total RNA master Panel II). Ethical approval for collecting cord blood, placental biopsies, brain samples and adult blood samples was granted by the ethical committee of Hospital Sant Joan de Déu Ethics Committee (study number 35/07) and IDIBELL (PR006/08 and PR048/13).

### Cell lines

Control lymphoblastoid cell lines were established by EBV transformation of peripheral blood cells and propagated as previously described [7]. Prior to ChIP, the lymphoblastoid methylation signature throughout the 8q24-imprinted domain was compared to leucocytes to ensure that the transformation process had not altered the epigenetic profile. The HEK293 cell line was used for the enhancer-blocking assay and was grown in DMEM supplemented with 10% FCS and antibiotics.

### Epigenetic bioinformatics analysis

RNA-seq datasets were downloaded from the NCBI GEO repository (GSM325476 and GSM 325483). Genomic data for H3K4me3 and corresponding meDIP in brain and non-neuronal samples used were retrieved from GSM54305, GSM613913, GSM772916, GSM772948, GSM772836, GSM669615, GSM669614, GSM806948, GSM806935 and GSM806943. The brain and leucocyte whole genome bisulphite sequencing datasets were from Lister *et al*. [32] (GSE47966, GSE46698 and GSE31263). The human CTCF, H3K4me1, H3K27ac and P300 ChIP-seq and ChIA-PET datasets were those with visual tracks on the UCSC genome browser (GRCh37/hg19) and raw reads were retrieved from GSM1022661, GSM1022662, GSM1022673, GSM651541, GSM772884, GSM772924, GSM772930, GSM772881, GSM772963, GSM772997, GSM670036, GSM772992, GSM772991, GSM670010, GSM77295, GSM773007, GSM670033, GSM772990, GSM670003, GSM772985, GSM772985 and GSM970215. Genomic mapping of all reads and data analysis was done with in-house scripts using the R statistical package.

### Allelic expression analysis

The placental and brain tissue genotypes of the expressed genes were obtained by PCR and direct sequencing. Sequences were interrogated using Sequencher v4.6 (Gene Codes Corporation, MI, Ann Arbor, USA) to distinguish heterozygous and homozygous samples. Heterozygous sample sets were analysed for allelic expression using RT-PCR that incorporated the polymorphisms in the final PCR product (Additional file 5: Table S1). The resulting RT-PCR amplicons were sequenced in both directions. The amplification cycle numbers for each transcript were determined to be within the exponential phase of the PCR, which varied for each gene, but was between 32 and 38 cycles.

### DNA-methylation analysis

Approximately 2 μg DNA was subjected to sodium bisulphite treatment and purified using the EZ GOLD methylation kit (ZYMO, Orange, CA, USA) and was used for bisulphite PCR analysis. Bisulphite PCR primers for each region were used with Hotstar Taq polymerase (Qiagen, Crawley, UK) at 40 cycles and the resulting PCR product cloned into pGEM-T easy vector (Promega, Madrid, Spain) for subsequent sequencing (Additional file 5: Table S1).

### Real-time RT-PCR

All PCR amplifications were run in triplicate on either Applied Biosystems 7500 or 7900 Fast real-time PCR machines (Applied Biosystems, Life Technologies, Grand Island, NY, USA) following the manufacturers’ protocol. All primers (Additional file 5: Table S1) were optimized using SYBR Green (Applied Biosystems, Life Technologies, Grand Island, NY, USA) and melt curve analysis to ensure that amplicons were specific and free of primer-dimer products. Thermal cycle parameters included Taq polymerase activation at 95°C for ten minutes for one cycle, repetitive denaturation at 95°C for 15 seconds, and annealing at 60°C for one minute for 40 cycles. All resulting triplicate cycle threshold (Ct) values had to be within one Ct of each other for the sample to be included. The quantitative values for each triplicate were determined as a ratio of *L19* expression, which was measured in the same sample, and the triplicate mean used to provide relative expression values.

### Pyrosequencing

Pyrosequencing was used as an accurate method of quantifying allelic expression of *PEG13* and *KCNK9* in heterozygous brain samples. Standard RT-PCR was used to amplify across SNPs with the exception that the reverse primers were biotinylated. The entire biotinylated RT-PCR product (diluted to 40 μl) was mixed with 38 μl of binding buffer and 2 μl (10 mg/ml) streptavidin-coated polystyrene beads. Bead-amplicon complexes were captured on a vacuum prep tool (Qiagen, Crawley, UK) and the PCR products denatured using 0.2 M NaOH. The denatured DNA was resuspended in 40 pmol of sequencing primer dissolved in 12 μl water and primers annealing was achieved by heating the sample to 80°C for two minutes before cooling to room temperature. For sequencing, forward primers were designed to the complementary strand (Additional file 5: Table S1). The pyrosequencing reaction was carried out on a PyroMark Q96 instrument (Qiagen, Crawley, UK). The peak heights were determined using the pyrosequencing commercial software. For methylation pyrosequencing of the *PEG13*-DMR, the same protocol was followed with the exception that the initial template was amplified from a bisulphite converted template and the interrogated sites were C/T variants at CpG dinucleotides.

### Chromatin immunoprecipitation (ChIP)

Chromatin from about 80 million cells was aliquoted into 100 mg batches and used for each immunoprecipitation reaction with Protein A Agarose/Salmon Sperm DNA (Millipore, 16-157, Billerica, MA, USA) and specific antibody. The antibody against CTCF (07-729) was obtained from Millipore (Billerica, MA, USA) and the antibodies against RAD21 (AB992) and SMC3 (AB9263) were obtained from Abcam (Cambridge, MA, USA). For each ChIP, a fraction of the input chromatin (1%) was also processed for DNA purification and a mock immunoprecipitation with a neutral, unrelated IgG antiserum was carried out in parallel [24].

Levels of immunoprecipitated chromatin at specific regions were determined by qPCR with an Applied Biosystems 7900 Fast real-time PCR machine, using SYBR Green (Applied Biosystems, Life Technologies, Grand Island, NY, USA). Each PCR was run in triplicate and protein binding was quantified as a percentage of total input material.

### Enhancer-blocker/Insulator assay

We used an enhancer-blocking assay to address the insulator activity of a DNA fragments containing the *PEG13*-DMR, *KCNK9* promoter and the ChIA-PET enhancer region utilizing the pELuc plasmid system [26]. The DNA fragments to be tested were cloned between the cytomegalovirus (CMV)enhancer and the promoter (Xho1) or upstream of the CMV enhancer (Pst1). The assay was performed by transfecting the constructs into HEK293 cells. The resulting data are presented as fold-enhancer-blocking activity normalized to the values achieved by the basal pELuc vector. As positive controls, we used the 5HS4 (1.2 kb chicken β-globin insulator) or the II/III (5′HS4 ‘core’ β-globin) insulator element [25]. For a negative control, we used II/III fragment with mutated CTCF binding sequence.

### 3C analysis

The chromatin conformation capture (3C) protocol was performed as previously described [39] with minor amendments. Approximately 100 mg of snap frozen brain tissue was reduced to powder with a pestle and mortar under liquid nitrogen. The pulverized brain samples were cross-linked with 1% formaldehyde for ten minutes and the reaction was blocked by adding glycine to a final concentration of 0.125 M. Subsequently, *Hind*III was used to digest 1 × 10^7^ formaldehyde cross-linked nuclei (overnight digestion, 1,200U, New England Biolabs, Ipswich, MA, USA ). The efficiency of the restricted enzyme digestion was assessed by qPCR across each restriction site, comparing digested and undigested chromatin fractions. Only chromatin with digestion efficiency above 80% was used. Subsequently, the DNA was ligated overnight in a 500 μl reaction volume using 1,950 units of T4 ligase (Fermentas- Fisher Scientific, Madrid, Spain). DNA was decross-linked by incubating overnight at 65°C and purified using phenol/chloroform extraction. This DNA was used for RT-PCR (LightCycler, Roche Applied Science, Barcelona, Spain) to determine the frequency of interactions, using constant primers either in the *KCNK9* promoter or the enhancer (Additional file 5: Table S1). Primer efficiency and basal interaction frequencies were determined using digested and ligated bacterial artificial chromosome (BAC) DNAs (human RPII 1069I18 and RPII 431 L20) as described by Braem and co-workers [39].

## Abbreviations

3C: chromosome conformation capture; 5-hmC: 5-hydroxymethylcytosine; ASD: autism-spectrum disorder; bp: base pair; BAC: bacterial artificial chromosome; ChIP: chromatin immunoprecipitation; CMV: cytomegalovirus; CpG: CpG dinucleotide; Ct: cycle threshold; DMEM: Dulbecco’s modified Eagle’s medium; DMR: differentially methylated region; ESTs: expressed sequence tags; FCS: fetal calf serum; H3K: histone 3 lysine; MR: mental retardation; ncRNA: non-coding RNA; qRT-PCR: real-time quantitative polymerase chain reaction; SNP: single nucleotide polymorphism; UCSC: University of California Santa Cruz; WGBS: whole genome bisulphite sequencing.

## Competing interests

The authors declare that they have no competing interests.

## Authors’ contributions

FC and CC: conception and design, data collection and analysis, manuscript writing and final approval of the manuscript. CVG, AGA, AS, DS, JS, ME, AMT, AR and LM: data collection and analysis and final approval of the manuscript. DM: conception and design, financial support, manuscript writing, final approval of manuscript. All authors read and approved the final manuscript.

## Supplementary Material

Additional file 1: Figure S1 **(A)** The expression of *TRAPPC9*, *PEG13* and *KCNK9* in a panel of human tissues as determined by qRT-PCR. All values are relative to the housekeeping gene *RPL19*. **(B)** The confirmation of *KCNK9* and *PEG13* allelic expression using pyrosequencing. Click here for file

Additional file 2: Table S2 The number of heterozygous tissue samples used to determine allelic expression of novel imprinted transcripts. Click here for file

Additional file 3: Figure S2 Determining the insulator activity of additional CTCF ChIA-PET regions. The bars indicate the firefly luciferase expression relative to *Renilla* luciferase activity for constructs containing a 370 bp fragment encompassing the CTCF within the *KCNK9* promoter or a 510 bp fragment containing the CTCF adjacent to the brain-specific enhancer. As a control, the enhancer-blocking assay was also performed with inserts for the 5HS4 (1.2 kb insulator), the II/III (‘core’ 5′HS4) of β-globin enhancer-blocker, as well as the II/III construct with mutated CTCF sites (II/III mut). The constructs are illustrated at the top of the figure. Data are presented as fold-enhancer-blocking activity normalised to the reference pELuc vector. The experiment represents the means of triplicate reading (±SD) with independent replicate experiments giving comparable results (data not shown). Click here for file

Additional file 4: Figure S3 The analysis of non-CG methylation within the gene bodies of *PEG13* and *AGO2*. **(A)** The CpG methylation (black) and CH methylation (grey) were determined from WGBS for grey and white matter. Only background levels of non-CG methylation were observed, equating to 1.3% and 0.7% at *PEG13* and 1.1% and 0.6% at *AGO2* in grey and white matter respectively. **(B)** Bisulphite sequence in frontal cortex derived DNA samples. The large circles represent single CpG dinucleotides whereas smaller circles depict individual CH on the strand, (●) a methylated cytosine, (O) unmethylated cytosines. Click here for file

Additional file 5: Table S1 Primers used in this study. Click here for file
