# Supplementary figures and images for: The PEG13-DMR and brain-specific enhancers dictate imprinted expression within the 8q24 intellectual disability risk locus
Source: Epigenetics Chromatin. 2014 Mar 25;7:5. doi: 10.1186/1756-8935-7-5 (PMC3986935; doi:10.1186/1756-8935-7-5)

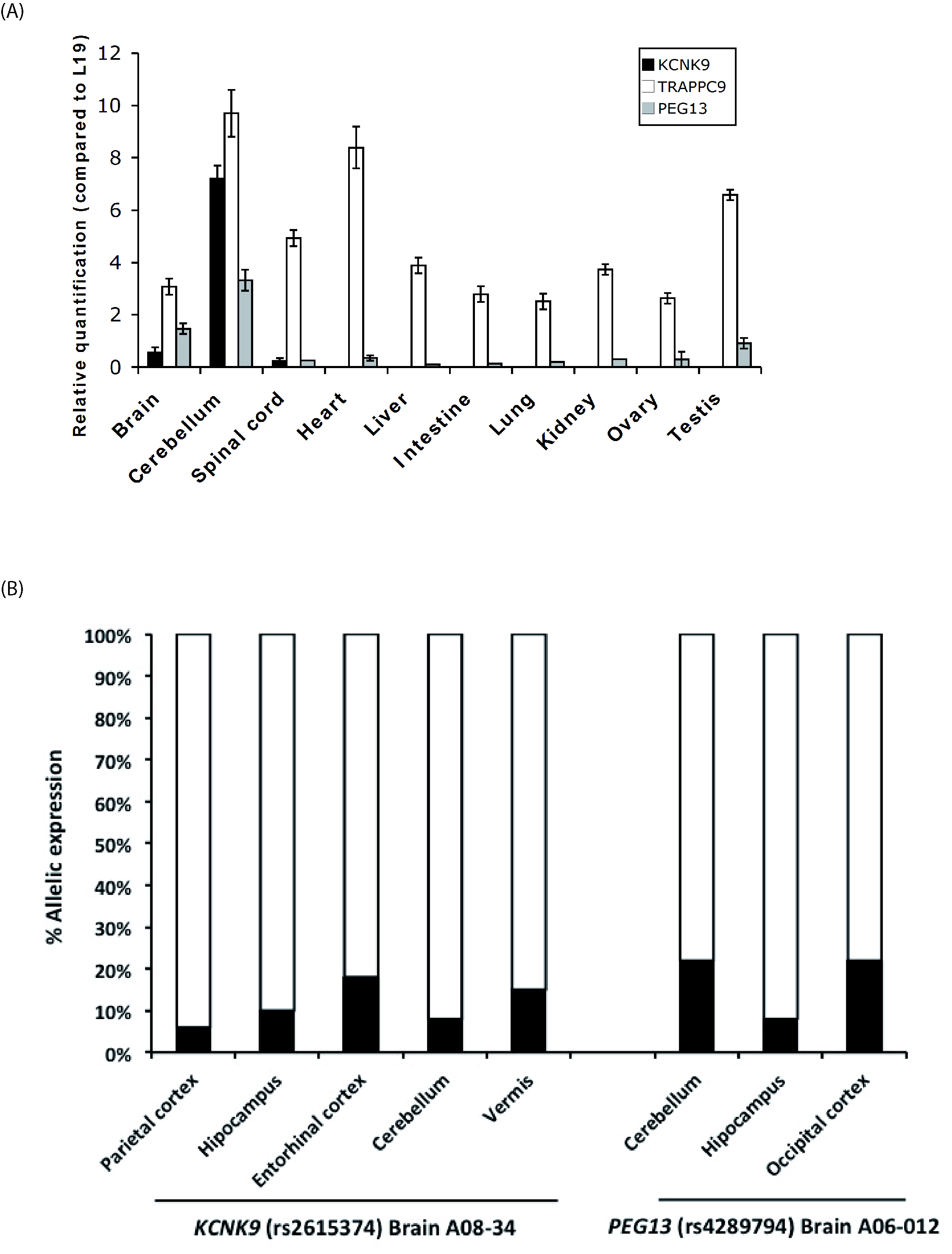

Supplement: Additional file 1: Figure S1 — (A) The expression of TRAPPC9, PEG13 and KCNK9 in a panel of human tissues as determined by qRT-PCR. All values are relative to the housekeeping gene RPL19. (B) The confirmation of KCNK9 and PEG13 allelic expression using pyrosequencing. [file 1756-8935-7-5-S1.tiff]

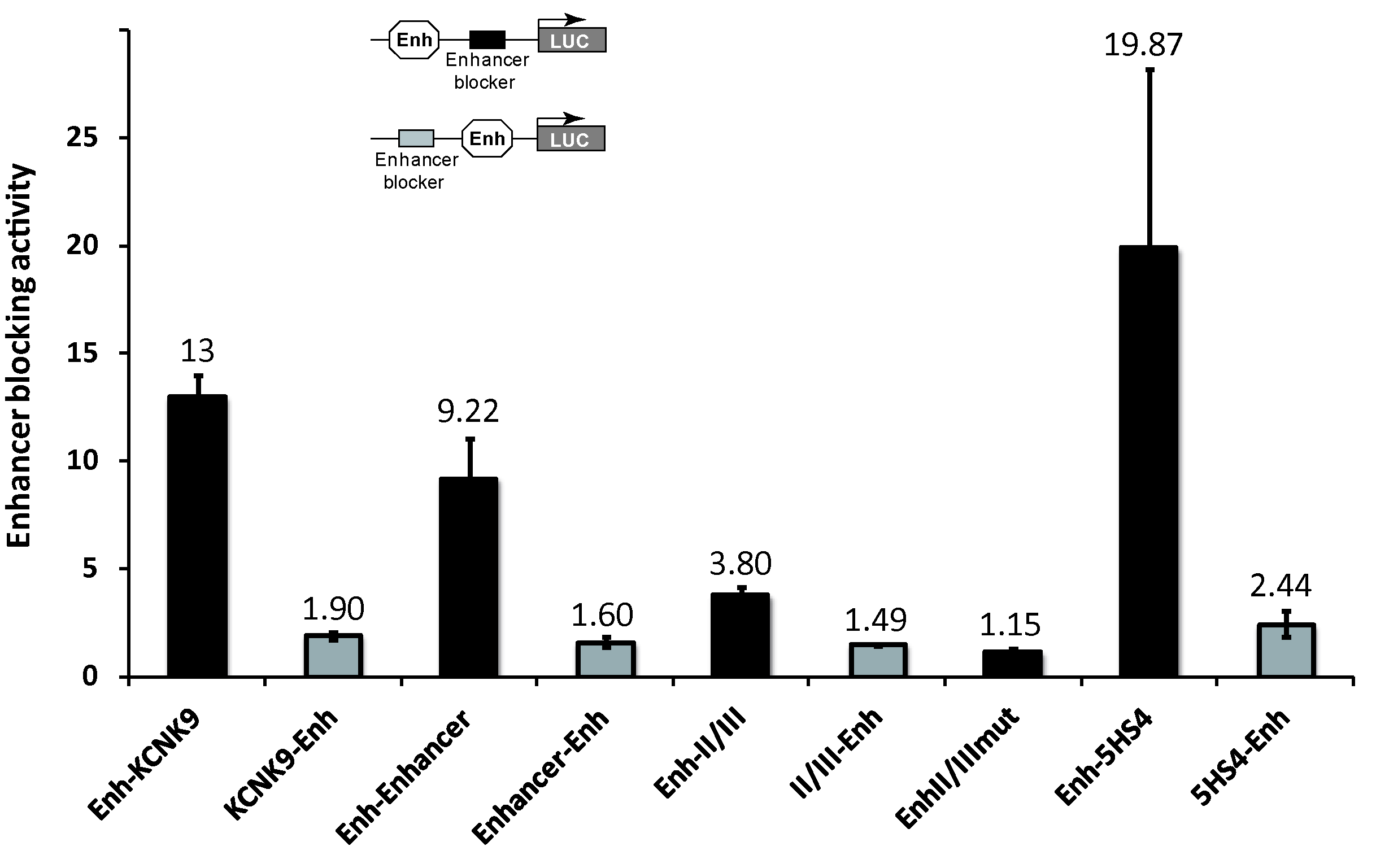

Supplement: Additional file 3: Figure S2 — Determining the insulator activity of additional CTCF ChIA-PET regions. The bars indicate the firefly luciferase expression relative to Renilla luciferase activity for constructs containing a 370 bp fragment encompassing the CTCF within the KCNK9 promoter or a 510 bp fragment containing the CTCF adjacent to the brain-specific enhancer. As a control, the enhancer-blocking assay was also performed with inserts for the 5HS4 (1.2 kb insulator), the II/III (‘core’ 5′HS4) of β-globin enhancer-blocker, as well as the II/III construct with mutated CTCF sites (II/III mut). The constructs are illustrated at the top of the figure. Data are presented as fold-enhancer-blocking activity normalised to the reference pELuc vector. The experiment represents the means of triplicate reading (±SD) with independent replicate experiments giving comparable results (data not shown). [file 1756-8935-7-5-S3.tiff]

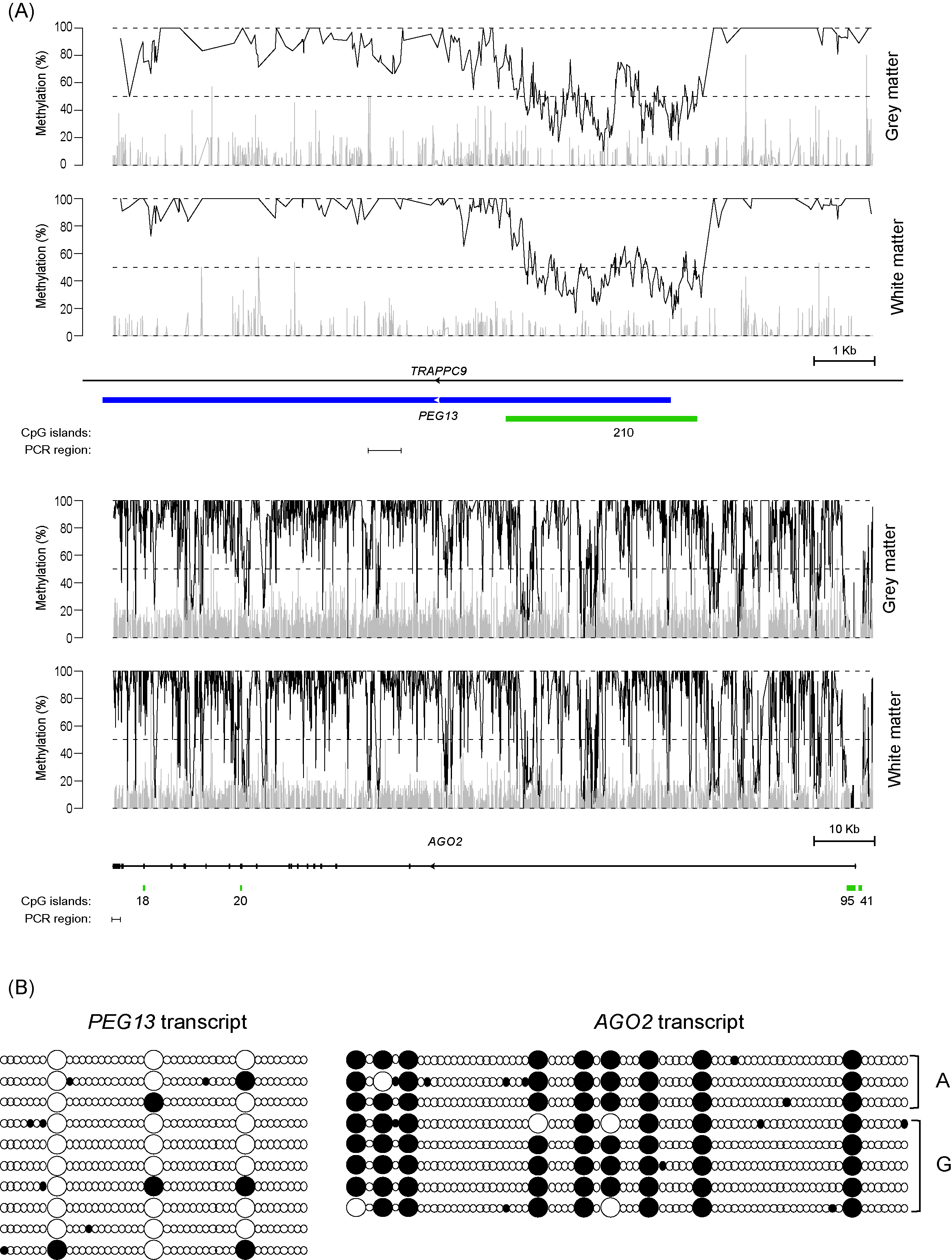

Supplement: Additional file 4: Figure S3 — The analysis of non-CG methylation within the gene bodies of PEG13 and AGO2. (A) The CpG methylation (black) and CH methylation (grey) were determined from WGBS for grey and white matter. Only background levels of non-CG methylation were observed, equating to 1.3% and 0.7% at PEG13 and 1.1% and 0.6% at AGO2 in grey and white matter respectively. (B) Bisulphite sequence in frontal cortex derived DNA samples. The large circles represent single CpG dinucleotides whereas smaller circles depict individual CH on the strand, (●) a methylated cytosine, (O) unmethylated cytosines. [file 1756-8935-7-5-S4.tiff]
