# Supplementary material for: The PEG13-DMR and brain-specific enhancers dictate imprinted expression within the 8q24 intellectual disability risk locus
Source: Epigenetics Chromatin. 2014 Mar 25;7:5. doi: 10.1186/1756-8935-7-5 (PMC3986935; doi:10.1186/1756-8935-7-5)
Supplement: Additional file 2: Table S2 — The number of heterozygous tissue samples used to determine allelic expression of novel imprinted transcripts. [file 1756-8935-7-5-S2.docx]

| **Gene** | **Number of heterozygous samples assessed by allelic RT-PCR** |
| --- | --- |
| *COL22A1* | Biallelic in 1 brain and 4 placenta samples. |
| *KCNK9* | 1 maternal expressed and 1 uniformative monoallelic brain samples. |
| *PEG13* | rs4289794- 1 paternally expressed and 9 monoallelic brain samples.  rs4455807- 1 paternally expressed and 6 monoallelic brain samples. |
| *TRAPPC9* | Biallelic in 1 leukocyte, 1 brain and 2 placenta samples. |
| *CHRAC1* | Biallelic in 2 leukocyte samples. |
| *AG02* | Biallelic in 1 leukocyte, 2 brain and 3 placenta samples. |

**Supplementary Table 2.** The number of heterozygous tissue samples used to determine allelic expression of imprinted transcripts.
