# Supplementary material for: The PEG13-DMR and brain-specific enhancers dictate imprinted expression within the 8q24 intellectual disability risk locus
Source: Epigenetics Chromatin. 2014 Mar 25;7:5. doi: 10.1186/1756-8935-7-5 (PMC3986935; doi:10.1186/1756-8935-7-5)
Supplement: Additional file 5: Table S1 — Primers used in this study. [file 1756-8935-7-5-S5.docx]

| **Gene** | **Primer sequence** | **SNP** |
| --- | --- | --- |
|  | **Genotypes and allelic RT-PCR** |  |
| COL22A1 | Geno F- GCCTCCCACCCCAGGCAGTGAACAG  Geno R- GAAAGCTTTCATCAGGACCTGGTATG  RT F- GCCTCCCACCCCAGGCAGTGAACAG  RT R- ATGCCTTTGCTGCCGTCTCTCCCA | rs10101430 |
| KCNK9 | Geno F- AGAGCCTGGGCGAGCGCATGAA  RT F- ACTTTGCGATCACGGTCATCACCA  Geno/RT R- GGTTGAGGAAGGCCCCGATGACC | rs2615374 |
| PEG13 | Geno/RT F- GTGCCGCAATCCACAGGGTACTGAC  Geno/RT R- GCTGAGACCTAGCTGGGTATC | rs4455807 |
| TRAPPC9 | Geno F- CATCCGGTTCCACGAGGAC  RT F- TATTTTTCACCCGAGTCAGCACCCTCC  Geno/RT R- TCTGCGTAGCCCAGCAAAGAT | rs199226 |
| CHRAC1 | Geno/RT F- AGTTTTAGAGCCCTCTATGGCTTTA  Geno/RT R- GAGCCAAACATTTACCCTAAG | rs4246131 |
| AGO2 | Geno/RT F- CCAGAATGCCTTCCGTCCCAG  Geno/RT R- ATGTTTGAGCCCATCAATTTCAT | rs1060832 |
|  | **qRT-PCR** |  |
| PEG13 | F- CGCAGCTGCAGGAGTGCCACAA  R- GCTGAGACCTAGCTGGGTATC |  |
| TRAPPC9 | F- TATTTTTCACCCGAGTCAGCACCCTCC  R- TCTGCGTAGCCCAGCAAAGAT |  |
| KCNK9 | RT F- ACTTTGCGATCACGGTCATCACCA  R- CAGTGTCAGCGGGATGCCCAGCAC |  |
|  | **ChIP** |  |
| Region 1 (KCNK9) | F- CAGCCCACGACGGAACCCCAGTCAGGC  R- ACAGCCTGGACTTCCCCGCAGCGA |  |
| Region 2 (PEG13-DMR) | F- CGCAGCTGCAGGAGTGCCACAA  R- GCTGAGACCTAGCTGGGTATC |  |
| Region 3  Enhancer | F- CAAAGGTCTGCCACCTACCTACCTCC  R- TCAGAAGACATTCTGGCATTATCT |  |
|  | **Bisulphite PCR** |  |
| PEG13-DMR | F- GTAGGGTATTGATGTTTACGTTGG  R- CTAAAACCTAACTAAATATCTCCCCTAC | rs4455807 |
| PEG13 transcript | F- GAATGAGTTGATATTGATGATAG  R- TTCCTACAACCTCCCTCTCCACT |  |
| KCNK9 promoter | F- GGTTTTTAGTTAGTTGGYGAGGTTGTTG  R- CAACCTACCATCTCTAACTAAATCC |  |
| AGO2 transcript | Out F- GTATTTTGTTTGATATGTTTTAGTG  In F- GTTATATTAGGATTAATAGATGGTTAG  R- TATTTTAAAACCCTAAATTCATAAACT | rs1060832 |
|  | **3C qPCR** | **Distance from constant (kb)** |
| Constant primer KCNK9 | cgctgctttctggccccaagc |  |
| KCN24F | ggaggcgatattggtcaagaac | 8499 |
| KCN29F | gaacccctctcaccctgtacc | 43311 |
| PE9R | ctttctgctcacagtctccttc | 326391 |
| PE10R | caggacaccttccaaggagtc | 332305 |
| PE20F | cacatggcctaatgcaagtatctg | 384616 |
| PE20R | cacagggaaaggtccttatcc | 384617 |
| PEG13 PROMF | ctgctgggatggtcattcaaagc | 398246 |
| PEG13 PROMR | gtactactcaaggagaaaaacagg | 398248 |
| PEG13 +2F | gaaaagaacatctttgggtagcc | 406113 |
| PE24R | gacaatgtgcatttgatgttgtatc | 409413 |
| PE27F | ccagacaactcctcactcacac | 423474 |
| PE29F | gtctcctccccttttggaaattctc | 435026 |
| PE30F | cctccaccttaatgagatggctc | 438371 |
| PE31F | gtgccacaaggccataaatagtcc | 440132 |
| 3R | gccatatgtaatatactatacgcaggtg | 445060 |
| PE36R | caggcggttaatttaagcactgg | 452432 |
|  |  |  |
| Constant primer Enhancer | gccatatgtaatatactatacgcaggtg |  |
| pe33R | GCAGACAAGGACTAATGAGTCC | -4418 |
| pe30F | CCTCCACCTTAATgAGATGGCTC | -6944 |
| pe29F | GTCTCCTCCCCTTTTGGAAATTCTC | -10204 |
| pe27F | ccagacaactcctcactcacac | -21851 |
| peg13+6f | GGTCCTCTCCATCCAGACAATC | -21881 |
| pe26R | GAACTCAATGCCTCTTCTGTACC | -30958 |
| pe24f | ACATAGgAAGTGAGTGAGCCTC | -35878 |
| peg13 +1f | gaaaagaacatctttgggtagcc | -41597 |
| pe22f | CATGGCAGGACTTGGTGGTATG | -41647 |
| peg13 promR | gacttgcagtctgtttctcttgc | -46977 |
| peg13 promF | ctgctgggatggtcattcaaagc | -47100 |
| pe20R | cacagggaaaggtccttatcc | -60470 |
| pe20F | cacatggcctaatgcaagtatctg | -60614 |
| pe19R | GCAGAGCTCCATGTTTCACTGC | -68519 |
| pe9R | ctttctgctcacagtctccttc | -118725 |
| pe4R | GTCTTGGCAGGAAAGGCTCTTAC | -130847 |
| KCN25R | GGCAGACATGGAAGCCTCACA | -431342 |
| KCN25F | ggtgatactatcagcactgaggc | -431483 |
| KCN23F | cagctgccaaggaagtgagtg | -441699 |
| KCNK9proR | CTTGAGTGGCCACCTACCAG | -445133 |
| KCNK9profbis | cgctgctttctggccccaagc | -445164 |
| KCN22F | atgtggagccagccctggaag | -445207 |
| KCN21F | gggaccagatgcttctgagttc | -460891 |
| KCN18F | caggtacagcaataagatcagggg | -466931 |
| KCN17F | gctcagggagacaccaggaaag | -469588 |
| KCN16F | catggcatctcccagacacaac | -488410 |

**Supplementary Table 1.** Primers sequences used in this study.
